# Comparative Assessment of Exchange-Correlation Functionals for the Structural, Thermodynamic, and Transport Properties of Liquid Lithium

**DOI:** 10.1021/acsomega.6c08835

**Published:** 2026-09-30

**Authors:** Romakanta Bhattarai, Thomas F. Fuerst, Stephen T. Lam

**Affiliations:** † Department of Chemical Engineering, 14710 University of Massachusetts Lowell, Lowell, Massachusetts 01854, United States; ‡ Tritium and Fusion Materials Department, 17212 Idaho National Laboratory, Idaho Falls, Idaho 83415, United States

## Abstract

Liquid lithium (Li) is an important material for advanced energy applications, particularly fusion energy systems, where it can serve as a tritium breeder, coolant, and plasma-facing material. The development of these technologies requires accurate predictions of liquid-state properties over a wide range of temperatures and operating conditions, making reliable first-principles simulations increasingly important. However, the performance and relative accuracy of commonly used density functional theory (DFT) exchange-correlation (XC) functionals for liquid Li have not been systematically benchmarked against experiment. In this work, we use ab initio molecular dynamics (AIMD) simulations to evaluate structural, thermodynamic, and transport properties of liquid Li. Structural properties probe local atomic ordering, thermodynamic properties, including density, bulk modulus, thermal expansion coefficient, and heat capacity, probe the liquid equation of state and thermal response, and self-diffusion probes atomic dynamics. Ten XC functionals are benchmarked, including local-density approximation (LDA), generalized gradient approximation (GGA), and zero-damping D3-corrected [D3(0)] functionals, and the results are systematically compared with available experimental data. The results show that the choice of XC functional has a significant impact on the predicted properties. At 1000 K, revPBE predicts an equilibrium volume of 25.00 Å^3^ per atom, compared with the experimental value of 25.09 Å^3^ per atom, whereas PBE-D3 predicts 22.06 Å^3^ per atom. The tested D3-corrected functionals generally predict smaller equilibrium volumes, higher densities and bulk moduli, and lower thermal expansion coefficients than experiment, indicating excessive binding within the tested D3-corrected framework. Among the functionals considered, revPBE provides the most balanced overall agreement with experiment, including a thermal expansion coefficient of 2.75 × 10^–4^ K^–1^ ± 8.24 × 10^–6^ K^–1^, compared with the experimental value of 2.77 × 10^–4^ ± 7.18 × 10^–6^ K^–1^, and a self-diffusion activation energy of 9.42 ± 2.03 kJmol^–1^, compared with 9.62 ± 1.26 kJmol^–1^. These results provide guidance for selecting experimentally anchored XC functionals for accurate AIMD modeling of liquid Li.

## Graphical abstract

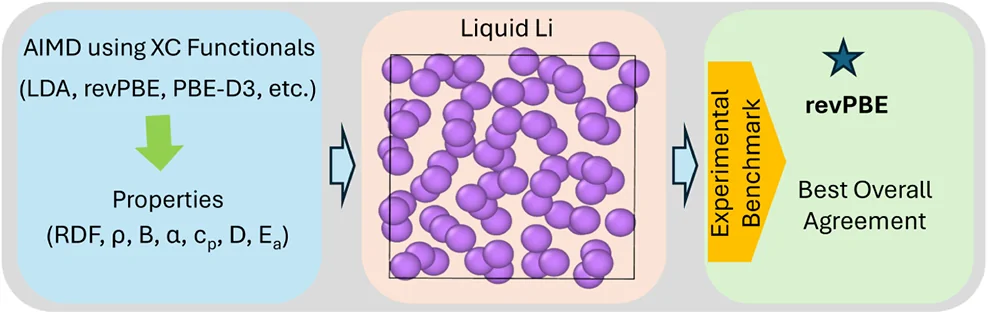

## Introduction

Liquid metals represent metals at high temperatures, i.e., in their molten state, and play a vital role in a wide range of technological applications, such as in tritium breeding blanket materials in fusion energy,^1^ coolant in advanced nuclear systems,^2^ energy storage devices,^3^ and reducing agents in molten salt extraction processes.^4^ Among them, liquid Li is one of the most interesting liquid metals because it combines very low atomic mass, high thermal conductivity, and unique transport behavior with strong sensitivity to temperature, pressure, and impurities. These properties make liquid Li a promising candidate for high-temperature heat transfer and advanced energy systems. The high absorption cross section for tritium production from Li-6 and neutron multiplication of Li-7 makes lithium an efficient tritium breeding material for fusion energy breeder blankets.^5^ However, its strong reactivity with air and water also makes it technologically challenging.^6^

A considerable amount of experimental work has been devoted to characterizing the properties of liquid Li. A NASA report published in 1968 provides reference data for several thermophysical properties, including density, heat capacity, thermal conductivity, and viscosity.^7^ Experimental studies have also investigated hydrogen and tritium transport in liquid Li because of their importance in fusion reactor applications.^8–11^ In addition, the self-diffusion coefficient of liquid Li has been measured using nuclear magnetic resonance techniques,^12^ while structural and dynamical properties have been examined using scattering experiments.^13^ More recently, Shu et al. studied liquid Li over a wide range of temperatures and pressures and reported pressure-induced changes in local atomic ordering.^14^

On the computational side, AIMD simulations have been performed to investigate the structural, electronic, and thermodynamic properties of liquid Li using different XC functionals. For example, Gonzalez et al. used the LDA functional to study the liquid–vapor interfaces and surface structure of Li and Na.^15^ Similarly, Yang et al. used PBE functional to investigate the effects of external pressure on the structural and electronic properties of liquid Li.^16^ To overcome the computational limitations of AIMD, several classical and machine-learning interatomic potentials (MLIPs) have also been developed, including 2NN-MEAM, SNAP, ReaxFF and PBE-trained MLIPs for Li.^17–20^

Recent machine-learning interatomic potentials have further expanded the accessible length and time scales for atomistic simulations of Li. MLIPs trained on DFT data have been developed to describe diverse Li environments, including bulk, surface, defect, and finite-temperature liquid configurations, while greatly reducing the computational cost relative to direct AIMD.^19^ More recently, an atomic-cluster-expansion potential for Li was trained on both ground-state DFT and finite-temperature AIMD data using PBE functional and was shown to describe solid and liquid phases, the melting point, viscosity, self-diffusion, and pressure–volume behavior.^21^ MLIP-based simulations have also been applied to liquid metallic systems and Li-containing alloys.^22^ However, MLIPs inherit the potential-energy surface represented in their reference data and therefore do not automatically eliminate systematic biases associated with the underlying XC functional. The choice of XC approximation used to generate the training data can consequently influence the transferability and predictive accuracy of the resulting MLIP. This consideration further motivates a systematic assessment of XC functionals for liquid Li.

The performance of XC functionals has been extensively assessed for a broad range of molecular, crystalline and condensed-phase systems.^23–28^ These studies provide useful qualitative guidance but do not provide a universal functional ranking, because XC-functional performance depends on the chemical system, thermodynamic state, and property of interest. In liquid metals, this dependence has been illustrated by benchmark studies of liquid Na. Han et al. compared LDA, PBE, PBEsol, and AM05 for liquid Na and evaluated pair correlations, equilibrium atomic volume, bulk modulus, and relative enthalpy at 600 and 1000 K.^29^ They found that the GGA functionals generally performed better than LDA, with PBE and AM05 giving the best overall agreement with available experimental data. In a separate study, Pozzo et al. calculated the electrical and thermal conductivities of liquid Na using LDA and GGA functionals and showed that the calculated conductivity is strongly sensitive to the simulated density.^30^

Despite these experimental and computational efforts, a systematic assessment of the performance of different XC functionals in predicting the structural, thermodynamic, and transport properties of liquid Li is still lacking. Previous AIMD studies on liquid Li generally used a single functional (LDA or PBE) to address specific questions, such as surface structure or pressure-induced structural changes, rather than to establish the relative accuracy of alternative XC approximations across multiple experimentally measurable liquid-state properties.^15,16^ Consequently, it remains unclear which commonly used XC functional provides the most balanced description of the density, local structure, compressibility, thermal expansion, heat capacity, and self-diffusion of liquid Li.

This issue is important because AIMD predictions of liquid-state properties can be sensitive to the XC approximation. A functional that yields a reasonable equilibrium density need not necessarily reproduce the transport behavior with similar accuracy. Therefore, a multiproperty comparison with available experimental data is required to identify the most balanced XC approximation for liquid Li. The issue is also relevant to the development of data-driven interatomic potentials, because the predictive quality of an MLIP is constrained by the accuracy and coverage of its reference electronic-structure data. Thus, identifying an XC approximation that reliably describes experimentally accessible properties of liquid Li is important both for direct AIMD simulations and for generating high-quality reference datasets for future data-driven modeling efforts.

In this work, we present a systematic AIMD benchmark study of liquid Li using ten commonly used XC functionals, including LDA, GGA, and functionals combined with the zero-damping D3-correction, denoted D3(0) (hereafter referred to as D3).^15,16,31–37^ Local and semilocal functionals, such as LDA and GGA, are routinely employed in simulations of liquid metals. In addition, dispersion-corrected functionals have improved structural and thermodynamic predictions for a variety of condensed-phase systems.^33,38–41^ However, their performance for liquid Li has not been systematically established. The present study is restricted to local, semilocal, and D3-corrected functionals that are computationally practical for statistically converged AIMD simulations of liquid Li. It is therefore not intended to provide a universal ranking of all available XC approximations, hybrid functionals, meta-GGA functionals, and alternative dispersion-damping schemes, which are outside the scope of the present work.^42–45^

We systematically investigate structural, thermodynamic, and transport properties, including density, bulk modulus, heat capacity, and diffusion properties. Together, these properties provide complementary validation of functional accuracy by probing local atomic ordering, equilibrium thermodynamics, and atomic dynamics. Accordingly, identifying an appropriate XC functional provides an important foundation for future development of MLIPs for large-scale simulations of liquid Li. By comparing the calculated results with available experimental data, we identify the XC approximation that provides the most balanced overall description of liquid Li within the tested functional set. We expect that this work will serve as a useful reference for selecting XC functionals for AIMD studies of liquid Li and for generating reference datasets for future MLIP development.

## Methods

AIMD simulations method is employed because it provides forces directly from the electronic structure and therefore allows the liquid structure and dynamics to be determined without introducing empirical interatomic potentials. This makes it suitable for predictive simulations of different properties and conditions. However, the AIMD simulations should be benchmarked as key approximations made in the exchange-correlations which balance accuracy and efficiency can exhibit sensitivity to system and chemistry. High temperature AIMD simulations of liquid Li are performed using VASP package,^46^ which uses pseudopotentials constructed under the projector augmented wave (PAW) method to describe the interaction between electrons and ion cores.^47^ We use ten different types of XC functionals: LDA,^48^ PBE,^49^ PBE-D3,^50^ PBEsol,^51^ PBEsol-D3,^50^ PW91,^52^ RPBE,^53^ RPBE-D3,^50^ revPBE,^54^ and revPBE-D3.^50^ The zero-damping vdW dispersion D3 as proposed by Grimme is also included for PBE, PBEsol, RPBE, and revPBE.^50^ A Gamma centered k-point mesh of 1 × 1 × 1 is used for the integration of Brillouin zone. A plane wave basis set with an energy cutoff of 700 eV is used for the expansion of wavefunction. The cutoff energy is chosen based on convergence tests (see Supporting Information for details). The electronic convergence criterion is set to 10^–5^ eV between any two successive self-consistent steps.^32,55^ The simulations are performed within the NVT ensemble for 50 ps with a time step of 2 fs in a cubical box with periodic boundary conditions consisting of 72 Li atoms at three different temperatures, ranging from 800 to 1200 K. The range of temperatures is chosen such that it lies well above the recommended melting temperature of liquid Li (453.7 K), and below its recommended normal boiling temperature (1608 K), based on the available thermophysical database.^7^ The liquid character is further supported by the calculated liquid-like structural and thermodynamic properties.

## Results and Discussion

The performance of the XC functionals is evaluated using structural, thermodynamic, and transport properties of liquid Li. Structural properties are assessed through the radial distribution function, while density, equilibrium volume, bulk modulus, thermal expansion coefficient, and heat capacity are used to evaluate thermodynamic behavior. Self-diffusion coefficients and activation energies are used to assess the predicted atomic dynamics. Together, these properties provide a quantitative comparison of the XC functionals for liquid Li.

### Equation of State and Equilibrium Thermodynamic Properties

Figure 1 shows the structure of liquid Li featuring 72 Li atoms in a cubical simulation cell of size 12.16 Å equilibrated at 1000 K using revPBE functional. We first determine the equilibrium volume of liquid Li by performing AIMD simulations at different cell volumes and fitting the resulting pressure–volume data to a third-order Birch-Murnaghan (B-M) isothermal equation of state,^56,57^ which is given by,

1 $$p\left(V\right)=\frac{3B_{0}}{2}\left[{\left(\frac{V_{0}}{V}\right)}^{7/3}-{\left(\frac{V_{0}}{V}\right)}^{5/3}\right]\left\{1+\frac{3}{4}\left(B_{0}^{’}-4\right)\left[{\left(\frac{V_{0}}{V}\right)}^{2/3}-1\right]\right\}$$

**Figure 1:**
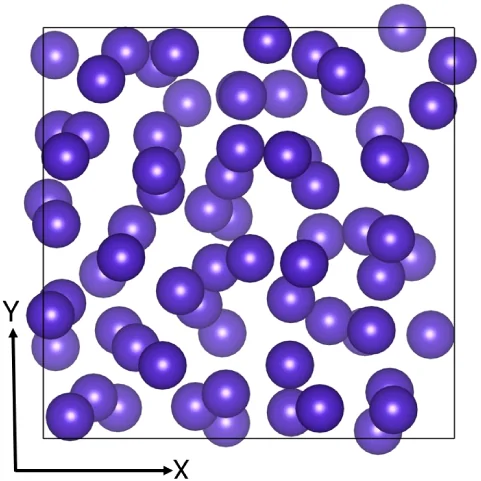
Structure of liquid Li at 1000 K simulated by revPBE functional.

Where, *P*(*V*) is the pressure at volume *V*, *V*_0_ represent the equilibrium volume (i.e., the volume corresponding to zero pressure), *B*_0_ = −V­(*∂_P_*/*∂_V_*)_*P*=0_ and $B_{0}^{’}={\left(\partial B/\partial P\right)}_{P=0}$ represent the bulk modulus and its pressure derivative at reference volume *V*_0_, respectively.

We fit the B-M equation of state [eq 1] to the AIMD results obtained at 800, 1000, and 1200 K to determine the equilibrium volume of the liquid Li, which is then used to determine the density and to assess the performance of the different XC functionals against experimental data. The fitting is done for aforementioned ten XC functionals.

The B-M equation of state curves for liquid Li at 1000 K are shown in Figure 2a. The experimental density of liquid Li at 1000 K is obtained by using the linear equation from NASA’s report^7^

2 ρ=562−0.1T

**Figure 2:**
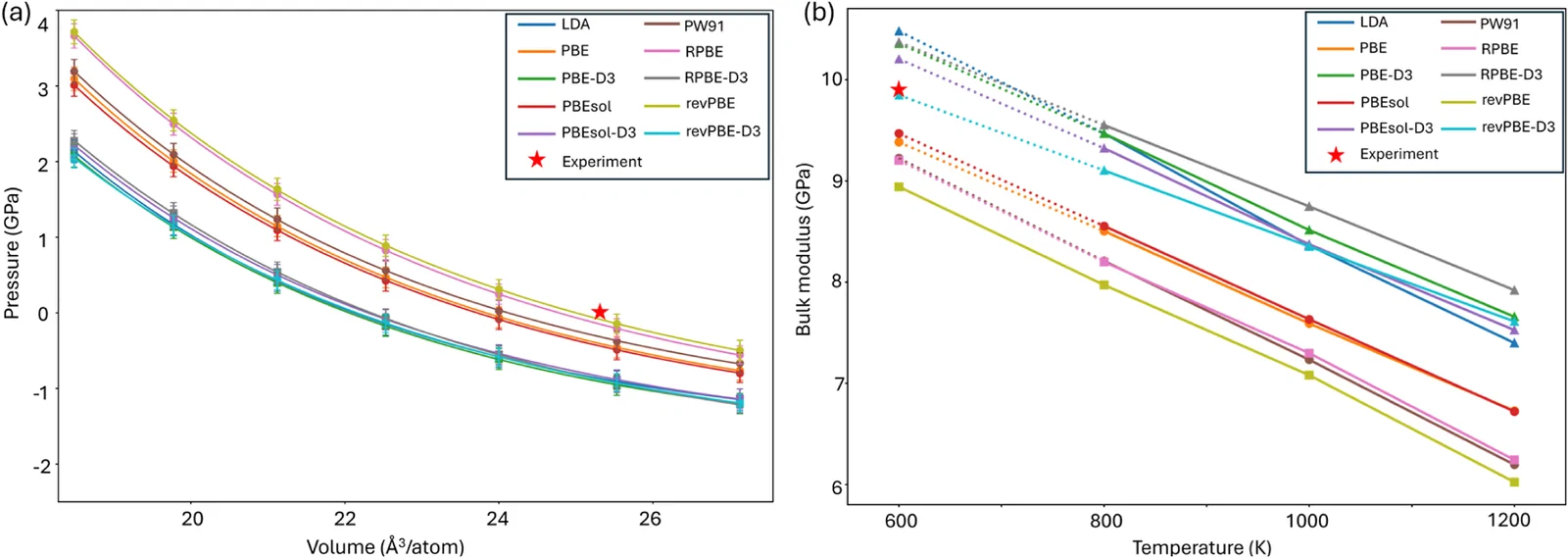
(a) Third-order B-M equation of state for liquid Li obtained from AIMD simulation at 1000 K using ten different XC functionals. (b) Calculated bulk modulus of liquid Li at different temperatures. The dotted lines represent linearly extrapolated lines. The red stars represent the corresponding experimental values in both panels.

Where *ρ* (kg/m^3^) is the density and *T*(*K*) is the temperature. The corresponding experimental volume (* symbol in Figure 2) is then calculated using the density-volume relation, and the corresponding simulation cell length is obtained as a cubic root of its volume.

Figure 2a shows that, among all functionals considered, revPBE functional predicts an equilibrium volume of liquid Li (25.00 Å^3^/atom), which is in close agreement with the experimental value (25.09 Å^3^/atom, as indicated by the red star) with only 0.36% deviation. The equation of state curves can be grouped into three clusters. The first cluster consists of revPBE and RPBE functionals, both of which predict the equilibrium volumes that are closest to the experimental volume. The second cluster includes PBE, PBEsol, and PW91, which systematically underestimate the equilibrium volume but remain reasonably close when compared with the experiment.

The third cluster comprises the D3-corrected functionals together with LDA, all of which predict significantly smaller equilibrium volumes than experiment. For example, the equilibrium volume predicted by PBE-D3 is 22.06 Å^3^/atom at 1000 K, which is approximately 12.08% smaller than the experimental volume. This underestimation is consistent with the additional attractive interactions introduced by the D3 correction, which favor more compact Li configurations and consequently increase the predicted density. Across the D3-corrected functionals considered here, the smaller equilibrium volumes, higher densities, and larger bulk moduli relative to experiment indicate a tendency toward overbinding in the present liquid-Li calculations. Given that liquid Li is a nearly-free-electron metal, where long-range interactions are strongly affected by electronic screening, the present results suggest that the inclusion of the D3 correction considered in this study does not improve the description of the equilibrium structure of liquid Li. However, establishing the microscopic origin of this behavior would require a more detailed analysis of the electronic structure and Li–Li correlations, which is beyond the scope of the present work. Similarly, LDA substantially underestimates the equilibrium volume, consistent with its well-known tendency to overbind, which leads to shorter interatomic distances, smaller equilibrium volumes, and larger bulk moduli in condensed systems.^51,58–60^ It is also noteworthy that the PBE functional which has been widely used in the simulation of liquid Li, underestimates the equilibrium volume by approximately 5%.^16,61^ Overall, the results indicate that revPBE and RPBE provide the most accurate description of liquid Li among the functionals examined in this work.

We extract the bulk modulus (*B*_0_) of liquid Li from eq 1 and compare its values across different XC functionals. The calculated *B*_0_ values for liquid Li obtained using ten XC functionals are shown in Figure 2b. For all functionals, the bulk modulus decreases approximately linearly with increasing temperature, reflecting the enhanced thermal motion and increased compressibility of the liquid at higher temperatures. Three distinct groups emerge in Figure 2b. The revPBE, RPBE, and PW91 functionals predict the lowest bulk moduli, whereas LDA and the D3-corrected functionals predict the highest values. PBE and PBEsol yield intermediate results. This trend closely matches the equilibrium volume predictions shown in Figure 2a.

The functionals that predict smaller equilibrium volumes generally yield larger bulk moduli, whereas those predicting larger volumes produce smaller bulk moduli. Among the functionals considered, revPBE predicts the lowest bulk modulus, whereas RPBE-D3 predicts the highest. For example, the bulk moduli obtained with revPBE are 7.97, 7.08, and 6.02 GPa at 800, 1000, and 1200 K, respectively, while RPBE-D3 predicts 9.55, 8.75, and 7.92 GPa at the same temperatures.

To further assess the performance of XC functionals against available experimental data, we examine the bulk modulus at 600 K, where experimental measurements have been reported. The corresponding values at 600 K are estimated by linearly extrapolating the temperature dependence of the calculated bulk moduli. In addition, an explicit AIMD simulation is carried out at 600 K for the revPBE functional, as it provides the closest agreement with the experimental equilibrium properties considered here. The predicted bulk modulus of revPBE is 8.94 GPa at 600 K, which is in good agreement with the experimental estimate of approximately 9.9 GPa reported within a pressure range of 8.7–11.5 GPa.^14^ This result is also consistent with previous theoretical studies.^19^ It should be noted, however, that the experimental bulk modulus was not obtained from direct measurements of the liquid volume. Instead, it was derived from diffraction data by assuming an fcc-like local structure above 8.7 GPa and fitting a second-order B–M equation of state over a relatively narrow pressure range (8.7–11.5 GPa) while fixing the pressure derivative of the bulk modulus. Therefore, the reported experimental value should be regarded as an approximate estimate derived from structural data. In contrast, the present AIMD results are obtained from equation-of-state fits over a broader pressure–volume range. Additional uncertainties may arise from finite-size effects in the simulations and from comparing the simulated 600 K state with the experimentally studied high-pressure liquid regime. Despite these differences, the revPBE bulk modulus of 8.94 GPa remains reasonably close to the reported experimental value of 9.9 GPa. We also compare our results with the bulk modulus of solid Li, which is approximately 10.43 GPa.^14^ The calculated bulk moduli of liquid Li are lower than this solid-state value over the temperature range considered.

Figure 3a shows the variation of equilibrium density of liquid Li predicted by ten XC functionals as a function of temperature. A nearly linear decrease in density is observed as the temperature increases from 800 to 1200 K. This trend is consistent with Figure 2a, where the equilibrium volume of liquid Li increases with temperature.

**Figure 3:**
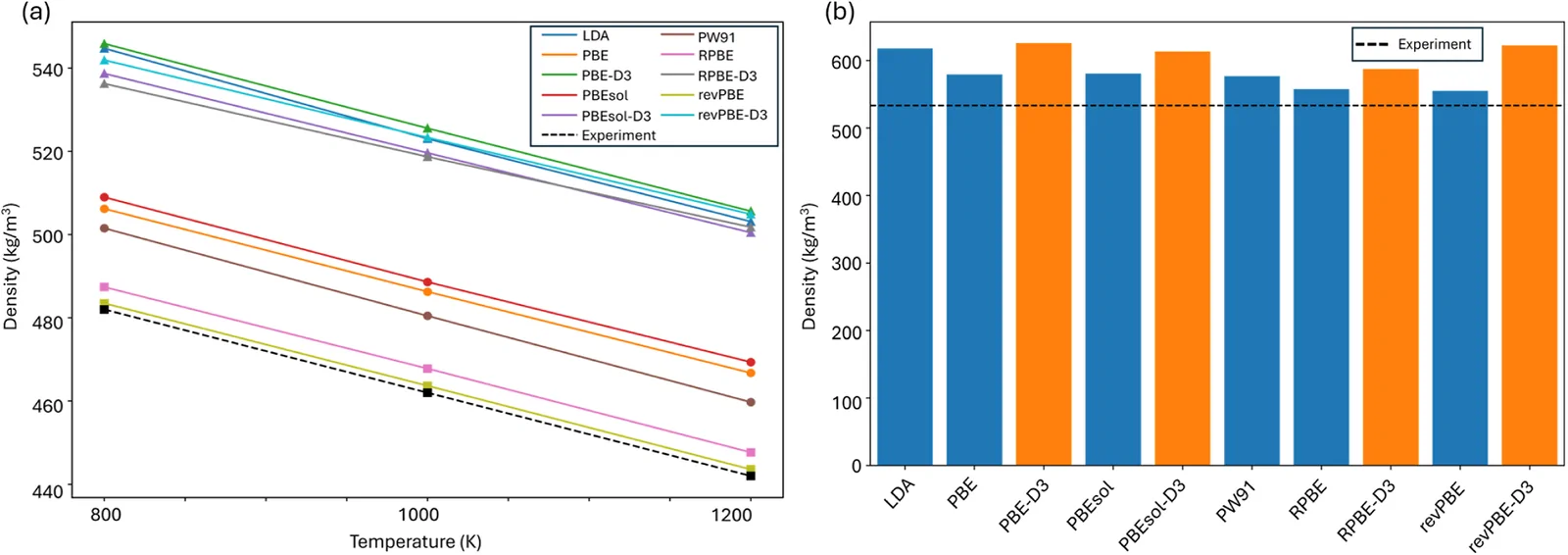
(a) Predicted density of liquid Li at different temperatures using ten different XC functionals (b) Corresponding density of solid Li at 0 K. The functionals with D3 correction are shown in orange color. Black dotted lines represent the experimental densities in both cases.

The predicted densities by revPBE are 483.49 ± 0.23, 463.67 ± 0.17, and 443.57 ± 0.43 kg/m^3^ at 800, 1000, and 1200 K, respectively. The corresponding experimental densities obtained from eq 2 are 482 ± 3.37, 462 ± 3.23, and 442 ± 3.09 kg/m^3^. Thus, revPBE reproduces the experimental density with an average deviation of approximately 0.35% over the temperature range considered. In contrast, the D3-corrected functionals generally predict higher densities. This behavior is consistent with their smaller equilibrium volumes, as reflected by the high-density cluster in Figure 3a.

To further examine the performance of revPBE, we extend our calculation to solid Li and investigate its density at 0 K by doing ground state DFT calculations with the same set of XC functionals. The calculated densities are shown in Figure 3b. The density of solid Li measured in the experiment^62^ is 533.40 kg/m^3^. Our calculations show that revPBE and RPBE functionals predict the densities of 554.88 and 557.73 kg/m^3^ respectively, which are approximately 4.03% and 4.56% higher than experimental value. Thus, both functionals provide a closer description of the experimental density than the widely used PBE functional, which predicts an equilibrium density of 579.38 kg/m^3^, approximately 8.62% higher than experiment. Similar to the liquid phase, the D3-corrected functionals generally predict higher densities than experiment, as shown in Figure 3b. Overall, among the functionals considered in this work, revPBE provides the closest agreement with the experimental densities of both liquid and solid Li.

Taken together with the smaller equilibrium volumes and larger bulk moduli discussed above, the higher densities predicted by the D3-corrected functionals are consistent with a tendency toward overbinding in the present calculations.

### Structural Properties

The structural properties are accessed through radial distribution functions (RDFs), which characterize the local atomic structure of liquid Li. The calculated RDFs of liquid Li at 1000 K using ten XC functionals are shown in Figure 4a. The RDF, represented by *g*(*r*), describes the probability of finding an atom at a distance *r* from a reference atom relative to an ideal gas of the same density. The RDF analysis is performed using AIMD trajectories generated at the equilibrium volumes predicted by the respective functionals (Figure 2).

**Figure 4:**
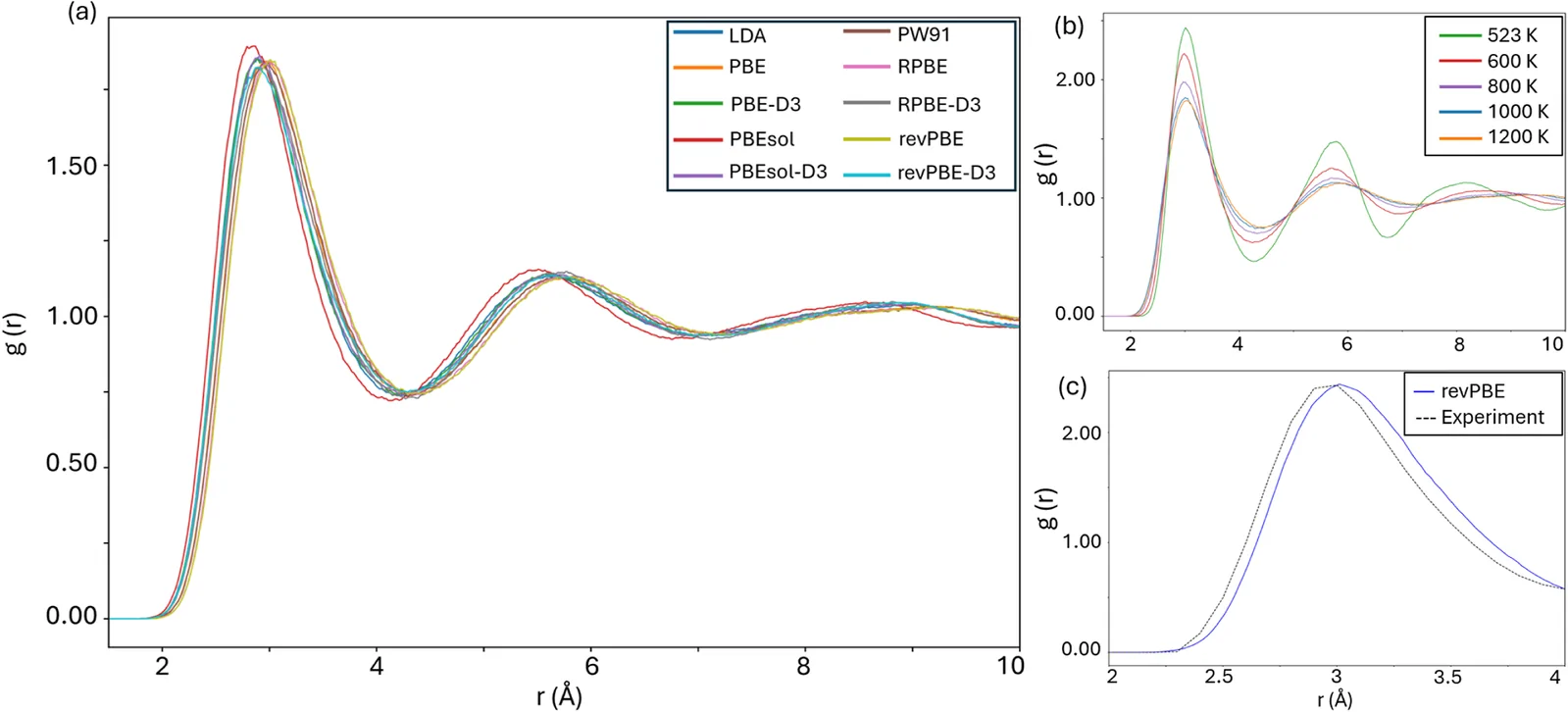
(a) RDF of liquid Li at 1000 K using ten different XC functionals (b) Evolution of RDF of liquid Li over the simulation temperature with the revPBE functional (c) Comparison of RDF obtained from the revPBE functional and experimental RDF at 523 K.^63^

As shown in Figure 4a, the overall RDF profiles predicted by the different functionals are remarkably similar, exhibiting nearly identical peak shapes and coordination-shell structures. However, a close inspection of the first RDF peak reveals a systematic dependence on the choice of XC functional. Among the functionals considered, PBEsol predicts the shortest nearest-neighbor distance, with the first peak located at 2.85 ± 0.02 Å. The D3-corrected functionals and LDA place the first peak near 2.90 ± 0.02 Å, whereas PBE predicts a slightly larger value of 2.97 ± 0.02 Å. The largest nearest-neighbor distances are obtained using PW91, RPBE, and revPBE, which predict the first peak at approximately 3.00 Å. These differences are consistent with the varying equilibrium volumes predicted by the different functionals.

Despite the differences in equilibrium volume among the XC functionals, the overall Li–Li RDF profiles remain similar, with the main differences occurring in the first-neighbor region. Thus, the present structural analysis does not establish a unique microscopic mechanism for the different thermodynamic properties. A direct connection between the D3-correction and changes in the electronic structure or effective Li–Li interactions would require additional electronic-structure analysis, which is beyond the scope of the present work.

To investigate the temperature dependence of the liquid structure, the RDFs calculated using the revPBE functional over the temperature range of 523–1200 K are presented in Figure 4b. As the temperature increases, the RDF peaks broaden and shift toward larger distances. Specifically, the first three prominent RDF peaks are located at 3.01 ± 0.02 Å, 5.79 ± 0.02 Å, and 8.15 ± 0.02 Å at 523 K, and shift to 3.03 ± 0.02 Å, 5.87 ± 0.02 Å, and 9.43 ± 0.02 Å, respectively, at 1200 K. The progressive broadening of the peaks indicates a broader distribution of Li–Li separations with increasing temperature.

Figure 4c compares the AIMD RDF obtained using revPBE at 523 K with the available experimental data from the Tohoku University liquid-metal database based on X-ray diffraction measurements.^63^ Good agreement is observed for both the position of the peak, *r*_max_ (expt: 3.00 ± 0.10 Å vs revPBE: 3.01 ± 0.02 Å) and its corresponding intensity, g­(*r*_max_) (2.43 vs 2.44). The overall RDF profile is reproduced well. Small difference between the simulated and experimental RDFs is within the resolution of the tabulated experimental RDF data and is comparable to uncertainties arising from RDF binning and finite-size effects in the AIMD simulations. Overall, the close agreement between the simulated and experimental RDFs indicates that the revPBE functional provides a good description of the local atomic structure of liquid Li at 523 K.

We calculate the coordination number (CN) of Li atoms in liquid Li. It is defined as,

3 $$CN=4\pi \rho \int_{0}^{r_{min}}r^{2}g\left(r\right)dr$$

Where, *ρ* is the number density of Li atoms, *g*(*r*) is the radial distribution function, and *r_min_* is the position of the first minimum of the RDF. Detailed information on the position of the first RDF peak, its corresponding intensity, the position of the first RDF minimum, and the coordination number of liquid Li at 1000 K obtained using the ten XC functionals considered in this study is summarized in Table 1. The coordination number is highest for PBEsol-D3 (14.06) and lowest for PBE (12.84), while revPBE predicts a CN value of 13.63. We also compare our results with the study of Krstic et al., who reported CN values for liquid Li obtained using the MEAM and ReaxFF potentials.^64^ The CN values predicted by the XC functionals considered in this work are generally higher than those obtained using these empirical potentials.

**Table 1:** Position of First RDF Peak [*r*_max_ (Å)], Corresponding Peak Intensity [g­(*r*_max_)], Position of First RDF Minimum [*r*_min_ (Å)], and Coordination Number of Liquid Li at 1000 K Using Ten XC Functionals Considered in This Study^a^

XC functional	r_max_ (Å)	g(r_max_)	r_min_ (Å)	CN
LDA	2.89 ± 0.02	1.86	4.21 ± 0.02	13.18
PBE	2.97 ± 0.02	1.84	4.37 ± 0.02	12.84
PBE-D3	2.89 ± 0.02	1.85	4.27 ± 0.02	13.72
PBEsol	2.85 ± 0.02	1.98	4.13 ± 0.02	13.92
PBEsol-D3	2.91 ± 0.02	1.86	4.25 ± 0.02	14.06
PW91	2.99 ± 0.02	1.84	4.33 ± 0.02	13.34
RPBE	2.99 ± 0.02	1.84	4.39 ± 0.02	13.28
RPBE-D3	2.95 ± 0.02	1.82	4.27 ± 0.02	13.23
revPBE	3.01 ± 0.02	1.85	4.29 ± 0.02	13.63
revPBE-D3	2.91 ± 0.02	1.82	4.29 ± 0.02	13.98
MEAM^64^	-	-	-	∼12.70
ReaxFF^64^	-	-	-	∼11.70
revPBE (523 K)	3.01 ± 0.02	2.44	4.27 ± 0.02	14.23
Expt (523 K)^63^	3.00 ± 0.10	2.43	4.10 ± 0.10	12.60

a Literature values obtained using MEAM and ReaxFF potentials, as well as experimental data and revPBE results at 523 K, are included for comparison.

Furthermore, we compare the CN predicted by revPBE with that derived from the available experimental RDF at 523 K. As shown in Table 1, revPBE reproduces the experimental position and intensity of the first RDF peak with good agreement. The calculated coordination number, however, is somewhat larger than the experimental value (14.23 vs 12.60). This difference originates primarily from the position of the first RDF minimum, which is predicted at a slightly larger distance by revPBE (4.27 Å) than observed experimentally (4.10 Å), indicating a broader first coordination shell. Since the coordination number is obtained by integrating 4*πρr* ^2^ *g*(*r*) up to *r*_min_, even small differences in the location of the first minimum can lead to noticeable variations in the resulting CN. Therefore, the larger CN predicted by revPBE is mainly associated with the broader first-shell boundary rather than differences in the first-neighbor peak.

The functional dependence of the coordination number indicates that the XC functionals produce modest differences in the local Li environment, although these differences do not by themselves establish a direct microscopic origin for the improved performance of revPBE.

### Temperature-Dependent Thermodynamic Properties

In addition to the equilibrium thermodynamic properties obtained from the equation of state analysis, temperature-dependent thermodynamic quantities such as the thermal expansion coefficient and heat capacity provide further insight into the behavior of liquid Li at finite temperatures. The volumetric thermal expansion coefficient, *α*, is calculated from the temperature dependence of the equilibrium volume and is defined as

4 $$\alpha =\alpha_{V}=\frac{1}{V}\left(\frac{\partial V}{\partial T}\right)$$

Where V and T are the volume and temperature of liquid Li, respectively. The calculated values of *α* for the ten XC functionals are shown in Figure 5a. The experimental value of *α* obtained from the density–temperature relation in NASA’s report^7^ is 2.77 × 10^–4^ ± 7.17 × 10^–6^ K^–1^.

**Figure 5:**
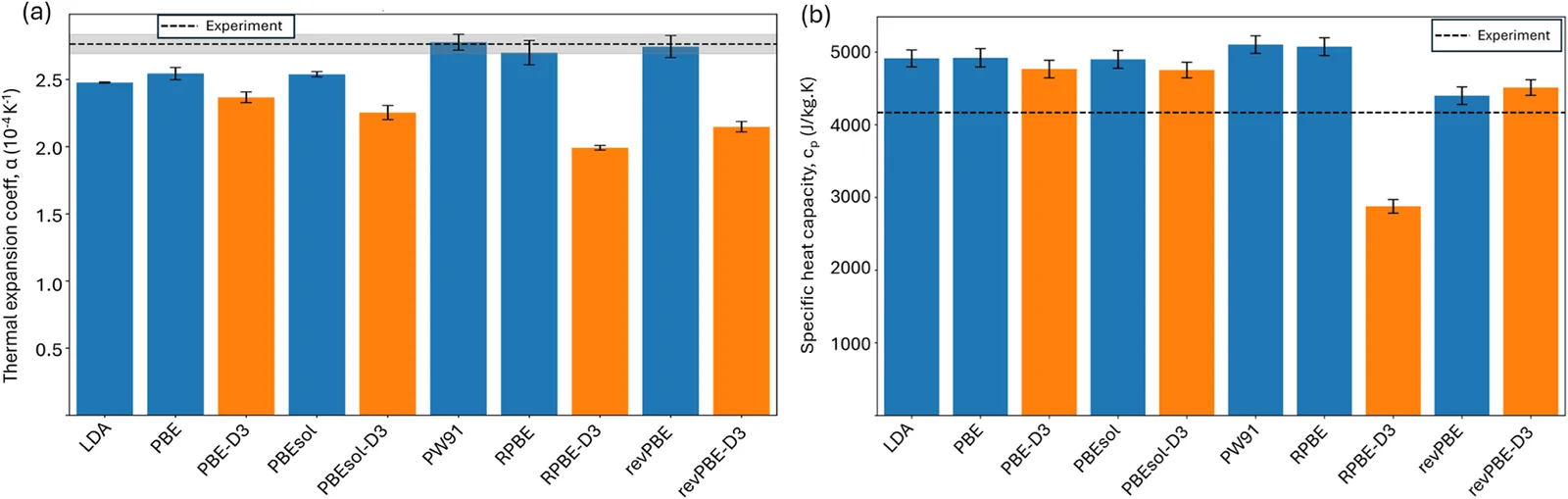
(a) Thermal expansion coefficient and (b) specific heat capacity of liquid Li calculated using ten different XC functionals. Black dotted lines represent the corresponding experimental values and the gray shaded region in (a) represents property uncertainty. The functionals with D3 correction are shown in orange color.

Among the functionals considered, we find that the *α* values predicted by PW91, RPBE, and revPBE functionals are in good agreement with experimental value within the uncertainty range. The corresponding values are 2.78 × 10^–4^ ± 5.95 × 10^–6^, 2.70 × 10^–4^ ± 9.10 × 10^–6^, and 2.75 × 10^–4^ ± 8.24 × 10^–6^ K^–1^, respectively, corresponding to deviations of approximately 0.72%, 2.17%, and 0.36% from the experimental value. In contrast, RPBE-D3 predicts the lowest thermal expansion coefficient (1.99 × 10^–4^ ± 1.75 × 10^–6^), with an approximately 27.90% deviation from the experiment.

A clear trend is observed in Figure 5a, i.e., functionals with D3 correction generally predict lower thermal expansion coefficients than the experimental value. This behavior is consistent with their tendency to predict smaller equilibrium volumes and higher densities. A similar reduction in thermal expansion due to stronger interatomic interactions has also been reported in layered materials such as graphite.^38^ The additional attractive interactions introduced by the D3 correction favor more compact configurations, which is consistent with the smaller equilibrium volumes and reduced temperature dependence of the volume observed in these calculations. As a result, the rate of volume expansion with increasing temperature $\left(\frac{\partial V}{\partial T}\right)$ becomes smaller, leading to lower values of *α*. These results indicate that the explicit D3 correction considered here doesn’t improve the description of the thermal expansion behavior of liquid Li and tend to favor a more compact liquid structure.

We also calculate the specific heat capacity of liquid Li using the same set of XC functionals. It is defined as

5 $$\begin{array}{l} C_{p}=C_{v}+VT\alpha^{2}B\\ C_{v}={\left(\frac{\partial U}{\partial T}\right)}_{V}\\ c_{p}=\frac{C_{p}}{m},{\;c}_{v}=C_{v}/m \end{array}$$

where *C_p_* and *C_v_* are the heat capacities at constant pressure and constant volume, respectively, *C_p_* and *C_v_* are the corresponding specific heat capacities, *m* is the mass of the simulation cell, *V* is the volume of the simulation cell, *T* is the temperature, *α* is the volumetric thermal expansion coefficient, *B* is the bulk modulus, and *U* is the internal energy. The quantity *C_v_* was estimated from the total internal energy obtained from AIMD simulations.

The specific heat capacity is calculated from the temperature dependence of the total energy and other previously obtained thermodynamic parameters, using eq 5, for the ten XC functionals. The results are shown in Figure 5b. The experimental value of *c_p_* of liquid Li according to NASA’s report^7^ is 4169 J kg^–1^K^–1^. As seen in Figure 5b, the predicted *c_p_* by revPBE, which is 4400.05 ± 119.95 J kg^–1^K^–1^, is closest to the experimental value among ten functionals with an approximate deviation of 5.54%. A similar pattern as in Figure 5a is also observed in Figure 5 (b) that the D3-corrected functionals systematically predict smaller value of *c_p_*, with RPBE-D3 being the lowest value (2877.34 ± 93.87 J kg^–1^K^–1^), except for revPBE-D3, which predicts a slightly higher value than revPBE, although within calculated uncertainties. The lower values of *c_p_* predicted by the D3-corrected functionals are consistent with their reduced thermal expansion coefficients and smaller equilibrium volumes. Since the contribution of thermal expansion to *c_p_* scales with *α* ^2^[eq 5], the lower values of *α* predicted by the D3-corrected functionals contribute to their lower values of *c_p_*. Differences in *c_v_*, *V*, and *B* also contribute to the calculated *c_p_*. Taken together, the thermal expansion and heat-capacity results indicate that revPBE provides the closest agreement with the available experimental values among the functionals examined for the temperature range considered in this work.

### Transport Properties

Finally, we access the transport properties of liquid Li. Transport properties such as self-diffusion characterize atomic mobility and provide a useful test of the interatomic interactions described by the simulations. We calculate the self-diffusion coefficient or diffusivity (*D*) of liquid Li using ten XC functionals. The diffusion coefficient reflects how rapidly Li atoms can move through the liquid and is calculated from the mean square displacement of Li atoms using the Einstein relation

6 $$D=\underset{t⃗\infty }{\lim}\frac{1}{6}\frac{d}{dt}\left\langle \right|r_{i}\left(t\right)-r_{i}\left(0\right){|}^{2}\rangle$$

Where ⟨|*r_i_* (*t*) – *r_i_* (0)|^2^⟩ is the mean square displacement of Li atoms, which is obtained from the AIMD trajectories after applying the periodic boundary condition unwrapping. The diffusion coefficients obtained at different temperatures are subsequently fitted using the Arrhenius relation

7 $$D=D_{0}\;\exp\left(-\frac{E_{a}}{RT}\right)$$

Where *D*_0_ is the pre-exponential factor (m^2^ s^–1^), *E*_a_ is the activation energy for diffusion (kJ mol^–1^), *R* is the ideal gas constant (8.3145 × 10^–3^ kJ mol^–1^ K^–1^). These parameters are obtained from the linear fit of ln *D* as a function of inverse temperature (1/*T*).

8 $$\ln\;D=\ln\;D_{0}-\frac{E_{a}}{RT}$$

Figure 6a shows the variation of diffusivity (*D*) for liquid Li as a function of (1/*T*) for the ten XC functionals. As expected from the Arrhenius relation, diffusivity decreases with increasing 1/*T*, corresponding to lower diffusion coefficients at lower temperatures. For comparison, the Arrhenius relations obtained from experiment and the MEAM potential are also shown as dotted black and red lines, respectively.

**Figure 6:**
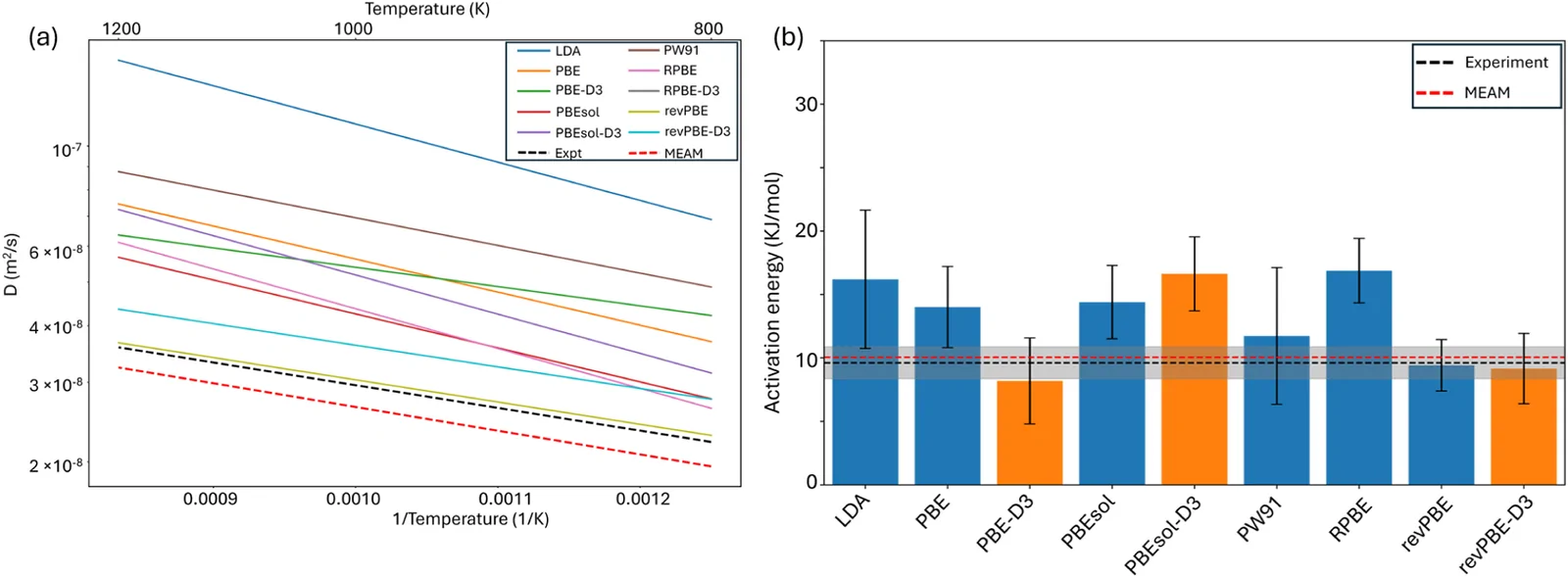
(a) Diffusivity (*D*) of liquid Li as a function of inverse temperature (1/T) plotted on a logarithmic scale, and (b) Activation energy of liquid Li calculated using ten different XC functionals. The black and red dotted line represent the experimental data and the results from MEAM potential, respectively.^18^ Error bars in panel (b) denote the uncertainty obtained from the linear regression used to fit the Arrhenius relation.

Among the functionals considered, revPBE provides the closest overall agreement with the experimental diffusion coefficients and reproduces the Arrhenius behavior reasonably well over the temperature range considered. The diffusion coefficients predicted by the MEAM potential are consistently lower than the experimental values. In contrast, RPBE-D3 exhibits a nonmonotonic temperature dependence and therefore does not follow the expected Arrhenius behavior. For this reason, RPBE-D3 is not shown in Figure 6.

In Figure 6b, we plot the activation energy (*E*_a_) of liquid Li for the ten XC functionals, which is obtained by fitting eq 7. The experimental value of *E*_a_ for liquid Li is 9.62 ± 1.26 kJ mol^–1^.^12^ In Figure 6b, it is evident that *E*_a_ is highly sensitive to the choice of XC functionals. Among the functionals considered, revPBE predicts an activation energy of 9.42 ± 2.03 kJ mol^–1^, which is in good agreement with the experimental value, with a deviation of approximately 2.08%. The MEAM potential gives a value of 10.05 kJ mol^–1^, corresponding to a deviation of about 4.47% from experiment.^18^ The relatively large uncertainties in the fitted *E*_a_ values reflect the sensitivity of the Arrhenius slope to uncertainties in the calculated diffusion coefficients. Longer trajectories and/or additional temperature points would reduce the statistical uncertainty and provide more robust estimates of *E*_a_ . Overall, revPBE provides the closest agreement with the experimental diffusion energetics among the functionals considered in this work.

The uncertainty in the extracted activation energies arises from the statistical uncertainty of the diffusion coefficients obtained from finite-length AIMD trajectories and the limited temperature window used in the Arrhenius fitting. The diffusion coefficient uncertainties are estimated using the standard error of the mean from block averaging and propagated through the Arrhenius fitting procedure. While the resulting uncertainties are relatively small, the differences observed among the XC functionals remain significant and the overall trends are preserved.

Taken together, the results suggest that the improved performance of revPBE is associated with a more balanced description of the equilibrium density and local structure of liquid Li, rather than with a substantial change in the overall Li–Li pair correlations. The similar RDF profiles obtained with the different XC functionals, together with the functional dependence of the coordination number, indicate that the XC functional modifies the local Li environment to some extent. These differences in the equilibrium structure and its response to temperature may contribute to the observed differences in thermal expansion, heat capacity, and self-diffusion. However, the present calculations do not allow us to establish a direct relationship between the observed property differences and changes in the electronic structure or effective Li–Li interactions. Such a microscopic interpretation would require additional analysis of the electronic structure and temperature-dependent interatomic interactions, which is beyond the scope of the present work.

Future studies could extend the present AIMD framework to hydrogen-containing lithium systems to investigate the effects of Li–H interactions and hydrogen bonding on the structural, thermodynamic, and transport properties of lithium-based materials. Recent studies of hydrogen-bonded systems have demonstrated the importance of hydrogen-bond strength, anharmonicity, vibrational coupling, and local molecular environments in determining their structural and spectroscopic behavior.^65–71^ Applying similar analyses to hydrogen-rich lithium-containing systems could provide insight into the relationships among Li–H interactions, hydrogen diffusion, and hydrogen uptake and release, with potential relevance to hydrogen-storage applications.

## Conclusions

In summary, we performed a systematic benchmark of ten DFT XC functionals to assess their performance in predicting the structural, thermodynamic, and transport properties of liquid Li. The results demonstrate that the choice of XC functional substantially influences the predicted properties. Among the functionals examined, revPBE provides the closest overall agreement with the available experimental data and offers the most balanced description of liquid Li across the structural, thermodynamic, and transport properties investigated. These results highlight the importance of carefully selecting XC functional for AIMD simulations of liquid Li and provide an experimentally anchored benchmark for future first-principles studies and dataset generation for MLIPs development. The D3-corrected functionals generally favor more compact liquid configurations and tend to overbind within the tested D3-corrected framework, resulting in poorer agreement with experiment for several equilibrium and thermodynamic properties considered in this work. Future studies may extend this benchmark approach to other liquid metals, metallic alloys, and alloys with solute impurities, which are relevant to advanced energy applications.

Although revPBE provides the closest overall agreement with the available experimental properties of pure liquid Li among the XC functionals examined in this work, this conclusion is specific to the thermodynamic and transport properties and temperature range investigated here. Its transferability to more complex environments relevant to fusion and hydrogen-energy applications, such as Li-containing hydrogen-rich systems, cannot be established from the present calculations alone. In such applications, Li–H interactions, chemical environments, pressure, and temperature conditions may alter the relative performance of different XC functionals. Therefore, additional benchmarking against experimental or higher-level theoretical data may be necessary to assess the suitability of revPBE under those conditions.

## Supplementary Material



## Data Availability

The data that support the findings of this study are available in Supporting Information.

## Notes

The VASP input files used in the AIMD simulations; DFT-calculated values of the density of liquid Li, density of solid Li, bulk modulus of liquid Li, thermal expansion coefficient of liquid Li, specific heat capacity of liquid Li, activation energy of liquid Li, and parameter D_0_ of liquid Li; and convergence plots of the plane-wave energy cutoff (PDF)
