# Supplementary material for: Comparative Assessment of Exchange-Correlation Functionals for the Structural, Thermodynamic, and Transport Properties of Liquid Lithium
Source: ACS Omega. 2026 Sep 30;11(40):62185–95. doi: 10.1021/acsomega.6c08835 (PMC13750380; doi:10.1021/acsomega.6c08835)
Supplement: Supplementary file 1 [file ao6c08835_si_001.pdf]

Supporting Information for

Comparative Assessment of Exchange-Correlation Functionals for the Structural, Thermodynamic, and Transport Properties of Liquid Lithium

Romakanta Bhattarai[1, *], Thomas F. Fuerst[2], and Stephen T. Lam[1, *]

[1]Department of Chemical Engineering, University of Massachusetts Lowell, Lowell, MA 01854, USA

[2]Tritium and Fusion Materials Department, Idaho National Laboratory, Idaho Falls, Idaho 83415, USA

[*]Corresponding author

Email: Romakanta_Bhattarai@uml.edu, Stephen_Lam@uml.edu

S1. VASP input files

The VASP input files which are used in the AIMD simulations of liquid Li at 800 K using revPBE functional are provided below. The VASP source code and the pseudopotential files are available on the VASP website that are distributed with license.

INCAR

PREC = Normal

ENCUT = 600

ISMEAR = 0

SIGMA = 0.05

NELM = 1000

EDIFF = 1e-5

IBRION = 0

ISIF = 2

ISPIN = 1

ISYM = 0

LCHARG = .FALSE.

```
LVTOT = .FALSE.
LWAVE = .FALSE.
LORBIT = 11
NCORE = 16
ALGO = Normal
LPLANE = .TRUE.
LSCALU = .FALSE.
LREAL = FALSE
NSIM = 4
TEBEG = 800
TEEND = 800
POTIM = 2
MDALGO = 2
NSW = 25000
SMASS = 0
GGA = RE
```

POSCAR

```
POSCAR_pure_Li
   1.00000000000000
    11.9960000000000000    0.0000000000000000    0.0000000000000000
     0.0000000000000000   11.9960000000000000    0.0000000000000000
     0.0000000000000000    0.0000000000000000   11.9960000000000000
   Li
    72
Direct
 -0.1676469104573491  0.3329579645339669  0.3131182676321753
```

2.0759640587335113	1.0127750458313973	0.8893788435305564
-0.0752194060398419	-0.4556749307683599	-1.0579617557649599
1.3971922412895050	-1.1259413310390551	-0.0157838199663870
-0.6359605462052084	0.7846410039648202	-0.3549717573894577
-0.2170175954503506	-0.3273541739104828	0.1892542216214320
0.2097820123703361	0.4093754306247218	0.9181336794564050
-1.3234393062600565	-0.6558119603226822	1.8570587862872738
-1.3254506012878131	-0.1540213632439447	0.9999627473733597
1.8602756821472799	-0.8615606289750146	1.0875776465656295
0.9563854256247388	-0.6850475184204402	0.8085664128127574
0.0950040508736689	0.7984028796354469	0.6755400911000853
1.4212962144748513	1.5954894666813408	1.3935623848046470
0.6124322413772686	0.2857270486571946	0.1377604221901293
1.1485918671397128	0.7963564244645865	1.3249249271892400
-0.0888324185324729	0.8753243319418620	-0.7051182161109445
-0.0958130654060556	0.1583498931122654	0.4457817117178798
0.6045408925543718	-0.2760723205359912	-0.6181880017703713
0.8329735121213868	2.0172449772325085	0.8472879507226861
0.7623031947762794	-0.8765691683313580	0.2814592823868531
0.0548248081846382	-1.3211396786927152	0.1558192362779460
-0.2494045085826757	1.4302185939145451	0.0273376274417259
0.9578335891092726	1.5238173650936155	-0.7084126429526846
1.2043313562087936	0.0457859548553394	0.6573652485259566
-0.3114822116556181	-0.1017053817160177	0.2172713730969569
2.0750606233314959	0.0664067486923818	1.1883817425401257
1.4161174811581121	0.6462859850677818	0.9326001984159724
-0.5549241453304116	1.2609483024843740	0.5904431468129147

0.4669436651254061	0.4069600416991623	-0.0345593507559953
-0.0978085858207252	-0.1827802566759256	-0.0065989736162879
0.6518704545630295	0.8978389196697294	-0.2875076166474881
0.8782168422087838	0.5513922260780129	0.7202373768499158
1.6300405272611052	1.5919000706985302	0.8613901923046861
0.3941402936093139	0.2311944575461786	0.8035566087696782
2.0123458808751171	-0.0502561128783936	-0.4664654357856494
0.6183715910445240	0.2443389482081046	1.4422726456489681
1.0944231450478774	1.2924959504494236	1.2917771539261844
1.2574482116576111	1.9032678848590561	0.5062875161664211
1.3557640669193554	-0.5163536423547748	-0.3493484477963469
0.7711279012906004	0.3535807701978292	0.5931453181329227
-0.8831708547482935	1.0314567413230789	1.3881162572744745
-0.1691431995647383	-0.2553415889386340	-0.5462786658517620
0.0622591802580618	0.2263689779111428	1.0080126241158445
0.2534612328804139	2.3107641222674182	0.4873027493312262
0.4852176527155991	0.7444594239282357	-1.8575776988893899
0.5575771675869229	0.1440658873885435	0.9538356392325066
0.3838260196363241	0.1056306847550265	1.4221572258440944
0.0530047826406769	-0.3607026836405212	1.4918626655742446
1.2347891110847384	0.5042925553077188	1.3572989422864636
1.0449349325968678	0.4435222857233068	1.1066796496662838
0.5687960457137045	1.4971161184284585	1.6756922460436237
1.5329686801270117	2.0044187458086888	0.1907535495425408
0.6341699032778680	1.6579558151921501	-0.4086092942678092
-0.3257632843243166	0.4963239467038558	1.3821281540273354
-0.4103272021497298	1.5294595350340612	-0.8711575428189194

-0.6916647119527239 1.0283240570874776 0.2385211229836452
1.1167585985230579 1.5388507493713737 0.7418878727798393
0.5225075285493143 0.9592849871615524 0.4472089917757695
0.3241535518593462 -1.7138604232831882 0.1523115002026557
1.3203868107879553 0.0842966782417325 1.9780913694524471
-1.1102214867190234 2.8254370432979394 0.6864724979698080
-0.5946176853006362 0.8441012577337310 0.3358910367804118
-0.3554299532202501 0.1536332274294659 -1.2889830778928664
2.1410577188906590 -0.2133844922104918 0.9295393942043556
1.2091416973382347 -0.1440325516116612 0.1205620943079410
2.0050175984044452 0.3915188825981288 0.5261195831015041
1.2466009054763583 1.5943451027093849 1.1250895456485477
-0.8175395451685731 -0.7411118728185824 1.6897601427692788
0.7271565956368086 0.0122229090756636 -0.5252709892796368
-1.1085172624872086 1.1689742168575692 0.6704295933778875
0.4668759886923226 0.3758475712960588 1.3409371277412485
0.3977927379093912 2.0216375115423046 -0.3192454470784585

KPOINTS

Kpoints

0

Gamma

1 1 1

0 0 0

S2. Density data of liquid Li (kg m^{-3})

temperature: [800, 1000, 1200],

LDA: [544.74 ± 0.03, 523.03 ± 0.24, 503.12 ± 0.41]

PBE: [506.20 ± 0.29, 486.28 ± 0.25, 466.75 ± 0.36]

PBE-D3: [545.86 ± 0.30, 525.54 ± 0.15, 505.61 ± 0.30]

PBEsol: [508.98 ± 0.20, 488.61 ± 0.13, 469.35 ± 0.32]

PBEsol-D3: [538.69 ± 0.08, 519.62 ± 0.31, 500.46 ± 0.27]

PW91: [501.51 ± 0.25, 480.46 ± 0.31, 459.74 ± 0.47]

RPBE: [487.39 ± 0.11, 467.80 ± 0.09, 447.66 ± 0.37]

RPBE-D3: [536.25 ± 0.21, 518.69 ± 0.29, 501.77 ± 0.46]

revPBE: [483.49 ± 0.23, 463.67 ± 0.17, 443.57 ± 0.43]

revPBE-D3: [541.91 ± 0.19, 523.32 ± 0.16, 504.90 ± 0.30]

Experiment: [482.00, 462.00, 442.00]

S3. Density data of solid Li (kg m^{-3})

temperature: [800, 1000, 1200]

LDA: 617.88

PBE: 579.38

PBE-D3: 625.78

PBEsol: 580.64

PBEsol-D3: 613.41

PW91: 576.69

RPBE: 557.73

RPBE-D3: 587.31

revPBE: 554.88

Experiment: 533.40

S4. Bulk modulus of liquid Li (GPa)

temperature: [800, 1000, 1200]

LDA: [9.47, 8.36, 7.40]

PBE: [8.51, 7.59, 6.73]

PBE-D3: [9.47, 8.52, 7.66]

PBEsol: [8.55, 7.63, 6.72]

PBEsol-D3: [9.32, 8.37, 7.53]

PW91: [8.21, 7.23, 6.20]

RPBE: [8.20, 7.30, 6.24]

RPBE-D3: [9.55, 8.75, 7.92]

revPBE: [7.97, 7.08, 6.02]

revPBE-D3: [9.10, 8.35, 7.61]

S5. Thermal expansion coefficient of liquid Li (K^{-1})

LDA: $2.48 \times 10^{-4} \pm 4.77 \times 10^{-7}$

PBE: $2.54 \times 10^{-4} \pm 4.63 \times 10^{-6}$

PBE-D3: $2.37 \times 10^{-4} \pm 4.01 \times 10^{-6}$

PBEsol: $2.54 \times 10^{-4} \pm 1.90 \times 10^{-6}$

PBEsol-D3: $2.26 \times 10^{-4} \pm 5.26 \times 10^{-6}$

PW91: $2.78 \times 10^{-4} \pm 5.95 \times 10^{-6}$

RPBE: $2.70 \times 10^{-4} \pm 9.10 \times 10^{-6}$

RPBE-D3: $1.99 \times 10^{-4} \pm 1.75 \times 10^{-6}$

revPBE: $2.75 \times 10^{-4} \pm 8.24 \times 10^{-6}$

revPBE-D3: $2.15 \times 10^{-4} \pm 3.89 \times 10^{-6}$

Experiment: $2.77 \times 10^{-4} \pm 7.18 \times 10^{-6}$

S6. Specific heat capacity of liquid Li ($J\ kg^{-1}\ K^{-1}$)

LDA: 4911.73 ± 115.75

PBE: 4920.95 ± 125.93

PBE-D3: 4766.43 ± 121.31

PBEsol: 4899.43 ± 121.82

PBEsol-D3: 4752.76 ± 107.53

PW91: 5102.52 ± 120.98

RPBE: 5074.69 ± 121.96

RPBE-D3: 2877.34 ± 93.87

revPBE: 4400.05 ± 119.95

revPBE-D3: 4510.74 ± 108.14

Experiment: 4169.00 ± 0.00

S7. Activation energy of liquid Li (kJ mol^{-1})

LDA: 16.20 ± 5.45

PBE: 14.01 ± 3.20

PBE-D3: 8.19 ± 3.38

PBEsol: 14.40 ± 2.89

PBEsol-D3: 16.63 ± 2.92

PW91: 11.74 ± 5.39

RPBE: 16.88 ± 2.54

revPBE: 9.42 ± 2.03

revPBE-D3: 9.16 ± 2.77

Experiment: 9.62 ± 1.26

S8. The value of parameter D_0 of liquid Li (m^{2}s^{-1})

LDA: (7.85 ± 4.36) × 10^{-7}

PBE: (3.03 ± 1.50) × 10^{-7}

PBE-D3: (1.44 ± 0.67) × 10^{-7}

PBEsol: (2.40 ± 0.73) × 10^{-7}

PBEsol-D3: $(3.83 \pm 1.10) \times 10^{-7}$

PW91: $(2.85 \pm 0.86) \times 10^{-7}$

RPBE: $(3.32 \pm 1.09) \times 10^{-7}$

revPBE: $(0.94 \pm 0.17) \times 10^{-7}$

revPBE-D3: $(1.09 \pm 0.31) \times 10^{-7}$

Experiment: $(0.94 \pm 0.40) \times 10^{-7}$

S9: Convergence plots of plane-wave energy cutoff (ENCUT):

Figure S1 shows the convergence of the calculated total energy of liquid Li with respect to the plane-wave energy cutoff (ENCUT) for different XC functionals. Calculations are performed for cutoff energies ranging from 300 to 1000 eV. The cutoff of 700 eV used in the AIMD simulations is chosen based on the convergence of the total energy.

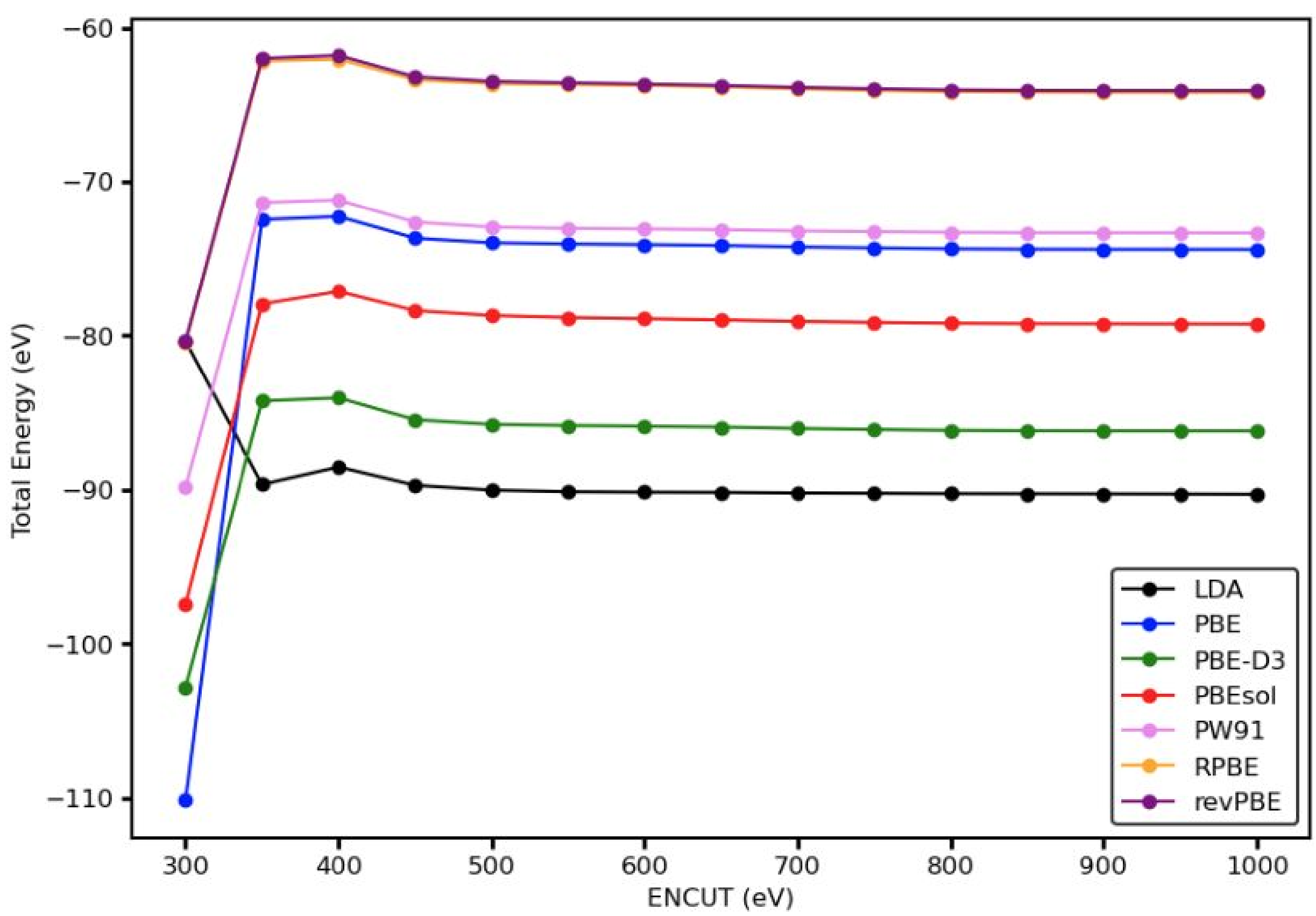

Figure S1. Convergence of the calculated total energy of liquid Li with respect to the plane-wave energy cutoff (ENCUT) for different XC functionals.
